# Protocol of the TREASURE study: Thoracic RadiothErapy with Atezolizumab in Small cell lUng canceR Extensive disease – a randomized, open-label, multicenter phase II trial

**DOI:** 10.1186/s12885-022-10074-9

**Published:** 2022-09-24

**Authors:** Farastuk Bozorgmehr, Petros Christopoulos, Inn Chung, Jelena Cvetkovic, Manuel Feißt, Johannes Krisam, Marc A. Schneider, Claus Peter Heußel, Michael Kreuter, Daniel W. Müller, Michael Thomas, Stefan Rieken

**Affiliations:** 1 Department of Thoracic Oncology, Thoraxklinik at University Hospital of Heidelberg, Röntgenstraße 1, 69126 Heidelberg, Germany; 2 Translational Lung Research Center Heidelberg TLRCH, Member of the German Center for Lung Research DZL, Im Neuenheimer Feld 156, 69120 Heidelberg, Germany; 3 University Hospital of Heidelberg, Institute of Medical Biometry, Im Neuenheimer Feld 130.3, 69120 Heidelberg, Germany; 4 Thoraxklinik at University Hospital of Heidelberg, Translational Research Unit (STF), Röntgenstraße 1, 69126 Heidelberg, Germany; 5 Thoraxklinik at University Hospital of Heidelberg, Diagnostic and Interventional Radiology with Nuclear Medicine, Röntgenstraße 1, 69126 Heidelberg, Germany; 6 Thoraxklinik at University Hospital of Heidelberg, Center for Interstitial and Rare Lung Diseases, Pneumology and Respiratory Care Medicine, Röntgenstraße 1, 69126 Heidelberg, Germany; 7 Institute of Clinical Cancer Research IKF GmbH at Northwest Hospital, Steinbacher Hohl 2-26, 60488 Frankfurt am Main, Germany; 8 Department of Radiation Oncology, University Medical Center Göttingen, Robert-Koch-Str. 40, 37075 Göttingen, Germany

**Keywords:** Extensive disease small cell lung cancer, SCLC, Radioimmuntherapy, Thoracic radiotherapy, Atezolizumab, Anti-PD-L1 monoclonal antibody, First-line therapy

## Abstract

**Background:**

Recently, the combination of the programmed death-ligand 1 (PD-L1) inhibitor atezolizumab with first-line chemotherapy has demonstrated to improve outcome for patients with advanced small cell lung cancer (SCLC), leading to approval of this regimen. At the same time, accumulating (pre-)clinical data suggest synergisms of radiotherapy and immunotherapy via the radiation-mediated induction of anti-tumor immunogenicity. Combining the recent findings, the TREASURE trial aims at further enhancing response to upfront chemo-immunotherapy by the addition of thoracic radiotherapy (TRT).

**Methods/design:**

The TREASURE trial is a randomized, multicenter, phase II clinical trial (ClinicalTrials.gov identifier, NCT04462276). One hundred four patients suffering from extensive disease (ED) SCLC, with any response to the standard of care induction chemo-immunotherapy will be randomized to receive atezolizumab maintenance therapy with or without TRT. The primary endpoint of this study is overall survival (OS). Secondary endpoints include further measures of efficacy, safety, and the collection of biomarker samples. A safety interim analysis will take place after *n* = 23 patients receiving TRT have been observed for three months after the end of TRT.

**Discussion:**

This trial will investigate whether treatment efficacy can be improved by adding TRT to atezolizumab maintenance therapy in ED SCLC patients with any response after chemo-immunotherapy. Safety and feasibility of such a regimen will be evaluated, and biomaterials for a translational research project will be collected. Together, the results of this trial will deepen our comprehension of how checkpoint inhibition and radiotherapy interact and contribute to the evolving landscape of SCLC therapy.

**Trial registration:**

Clinicaltrials.gov identifier: NCT04462276 (Date of initial registration: 8th July 2020), https://clinicaltrials.gov/ct2/show/NCT04462276

Eudra-CT Number: 2019-003916-29 (Date of initial registration: 30th March 2020), https://www.clinicaltrialsregister.eu/ctr-search/trial/2019-003916-29/DE

## Background

With around 12 -15% of lung cancer cases worldwide, small cell lung cancer (SCLC) is one of the main causes of cancer-related mortality [1]. This aggressive tumor entity frequently shows early development of widespread metastases. Thus, the majority of patients present at a stage of extensive disease (ED), and it is of major importance to develop strategies to improve the outcome of these patients.

One approach that has been explored in the past is the addition of thoracic radiotherapy (TRT) to chemotherapy. In a phase III randomized trial, Slotman et al demonstrated an increased 2-year survival rate with low toxicity rates for patients who received TRT (30 Gray [Gy] in 10 fractions) in addition to prophylactic cranial irradiation (PCI) after any response towards standard chemotherapy, compared to patients who did not receive TRT [2]. Progression was less likely in the TRT group (hazard ratio [HR] = 0.73, *p* = 0.001) with an almost 50% reduction in intrathoracic recurrences. The randomized phase II RTOG-0937 trial was designed to evaluate PCI ± consolidative RT to intrathoracic disease and limited extracranial metastases in ED-SCLC patients. Results of RTOG-0937 showed that consolidative RT prolonged progression free survival (PFS), but did not improve 1-year overall survival (OS) [3]. Since both studies provide interesting signals for a potential benefit of local thoracic consolidation, but failed to reach statistical significance within their trial designs, a controversial discussion about who might profit from thoracic radiotherapy is ongoing. Based on German guidelines (Onkopedia and consultation version of new S3-guideline), thoracic radiation can be offered to patients with residual disease following systemic treatment on an individual basis. This is in line with the ESMO guidelines, which mention consolidation radiotherapy to the residual tumor and lymph nodes as a treatment option (grade C, level II recommendation) for patients with a performance score of 0-2 who achieve a response after chemotherapy, while the ASTRO guidelines strongly recommend thoracic radiotherapy for patients with a response to chemotherapy alone but residual tumor in the thorax. At the same time, all guidelines stress that there is a lack of data considering the implementation of consolidation thoracic radiotherapy to immunochemotherapy in ED-SCLC. The advent of immunotherapies has revolutionized the management of several tumor entities, including advanced non-small cell lung cancer (NSCLC). Here, upfront mono- or combination therapies with immune checkpoint inhibitors (ICI) are now standard of care in the majority of patients. Considering their high mutational burden, SCLC tumors were also expected to respond well to this type of treatment. Indeed, results from several clinical trials suggest that SCLC patients may benefit from such therapies [4, 5]. In particular, the addition of a programmed death-ligand 1 (PD-L1) checkpoint inhibitor to chemotherapy in order to enhance T cell-mediated immunity appears to be very promising, as was demonstrated by the results of the IMpower133 trial [6]. Here, adding the PD-L1 antibody atezolizumab to carboplatin/etoposide induction therapy followed by an atezolizumab maintenance therapy significantly prolonged OS and PFS compared to the placebo arm. Results of the CASPIAN phase 3 study, where combination of platinum-etoposide with durvalumab in first-line treatment for patients with ED-SCLC resulted in stable and persistent clinical benefit across OS, PFS, and objective response [7], align with Impower133 trial findings. Addition of pembrolizumab (anti-PD-1) to etoposide and platinum treatment, as part of the KEYNOTE 604 study, significantly prolonged PFS in comparison with placebo plus etoposide and platinum as first-line therapy for patients with ED-SCLC [8]. Despite these encouraging data on outcome, the question remains how the response can be further increased.

Along this line, the combination of photon radiotherapy with immunotherapeutic approaches has recently caught attention as an attractive approach for treatment of lung cancer. The rationale behind this lies in the capability of radiotherapy itself to promote immune-mediated anti-tumor responses through a variety of processes, such as inducing the immunogenic death of tumor cells resulting in antigen release and broadening of the immune repertoire of T cells, the enhancement of chemokine-mediated T cell recruitment towards the irradiated tumor, or the upregulation of major histocompatibility complex I (MHC I) and tumor-associated antigens that increase tumor cell vulnerability [9]. The radiation-induced effects on the microenvironment and the activation of immune cells can result in tumor regression even distant from the irradiated field, a phenomenon that has been first observed in the 1950s and is referred to as the “abscopal effect” [10]. Several clinical trials started to explore the combination of ICI and radiotherapy. In unresectable NSCLC stage III, the PACIFIC trial has proven an immense clinical impact of treatment with durvalumab after chemoradiotherapy with notably low toxicities resulting in the approval of this regimen [11, 12]. In the phase II DETERRED trial, concurrent atezolizumab administration with chemoradiation therapy (CRT) followed by consolidation and maintenance atezolizumab did not show increased toxicities compared to CRT alone followed by consolidation and maintenance atezolizumab [13]. Furthermore, recent analyses have also suggested a good tolerability of the combination of immunotherapeutic drugs and radiotherapy in patients with different solid tumors [14–16].

The TREASURE trial presented here combines the IMpower 133 regimen with thoracic radiotherapy and will explore efficacy and feasibility of this treatment. The objectives of this trial are to: i.) increase the efficacy of chemotherapy combined with atezolizumab by adding radiotherapy, ii.) assess the safety and tolerability of the combination of chemotherapeutic, immunological and radiological treatment in the first-line setting of advanced SCLC, and iii.) collect tumor tissue, blood and stool samples for translational research.

## Methods/design

### Study design, setting, objectives, and characteristics of participants

The TREASURE trial is an open-label, randomized, multicenter phase II clinical trial to examine the feasibility and treatment efficacy of TRT with the IMpower133 regimen in first-line treatment of patients with advanced SCLC. The main objective is the increase of efficacy of atezolizumab maintenance after induction with chemo-immunotherapy by adding TRT. Secondary objectives include the determination of safety and tolerability of radiological and immunological treatment in the first-line setting of ED SCLC and the prospective biomaterial collection for an accompanying translational research program. This study will be performed in compliance with the Declaration of Helsinki principles, and the protocol was accepted by the responsible institutional review boards.

In total, one hundred four patients are planned to be included in twenty sites across Germany and Austria within 24 months. Clinicaltrials.gov (NCT04462276) provides access to a complete list of sites. Recruitment began in September 2020 (First Patient In) and will be finalized in July 2024.

**Fig. 1:**
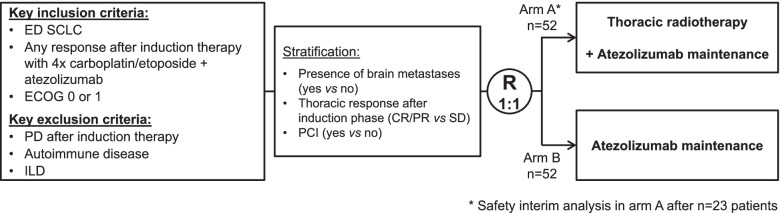
TREASURE study design. ED SCLC patients with any response (defined as CR/PR or thoracic SD with CR/PR of extrathoracic lesions) after four cycles of standard chemo-immunotherapy consisting of carboplatin/etoposide and atezolizumab will be randomized to either receive thoracic radiotherapy or not. All patients will receive maintenance atezolizumab therapy until disease progression or occurrence of intolerable toxicities

Upon arrival at the clinic, patients who may be eligible for study inclusion will be approached and invited to participate. At time of inclusion into the study, all patients must have been diagnosed with ED SCLC and responded to four cycles of induction therapy with carboplatin/ etoposide and atezolizumab, which they have received independently of the study as part of standard of care therapy (Fig. 1). Before study enrolment, all documents and their imaging files will be reviewed by a board-certified radiation oncologist, as to whether or not the thoracic tumor formation can safely be treated by thoracic radiotherapy – based upon the expected target coverage and sparing of critical organs, such as the lungs. The main eligibility criteria are specified in Table 1.

**Table 1:** Key inclusion and exclusion criteria of the TREASURE trial

Inclusion criteria	Exclusion criteria
• Fully-informed written consent • Confirmed ED SCLC • ECOG performance status score ≤ 1 • Any response after four cycles of induction chemo-immunotherapy defined as CR/PR or thoracic SD with CR/PR of extrathoracic lesions • Thoracic treatment volume considered treatable using acceptable radiation fields as judged by a radiation oncologist • 28 ± 7 days between last administration of chemo-immunotherapy and randomization. • Patients with a history of treated CNS metastases are eligible, if there is no ongoing requirement for corticosteroids as therapy for CNS disease. Patients with asymptomatic brain metastases that do not require local therapy with irradiation (whole brain irradiation) can be included. • No previous radiotherapy to lung and mediastinal lymph nodes within the past 5 years • Availability of pre-treatment tumor tissue specimen • FEV1 ≥ 40% • Adequate bone marrow, renal function, and hepatic functions	• Prior treatment with immunotherapeutic drugs (with the exception of induction chemo-immunotherapy as defined in inclusion criteria) • Prior therapy for limited-stage SCLC with curative intent • History or current radiology suggestive of interstitial lung disease (ILD) (including but not limited to idiopathic pulmonary fibrosis (IPF), formerly described as usual interstitial pneumonia (UIP), and cryptogenic fibrosing alveolitis (CFA)), non-infectious pneumonitis, drug-induced pneumonitis, idiopathic pneumonitis. • Any concurrent cancer treatment or major surgery (as defined by the Investigator) • Active or prior documented autoimmune or inflammatory disorders or history of active primary immunodeficiency • Current use of immunosuppressive medication • Positive testing for hepatitis B virus surface antigen (HBV sAg), hepatitis C virus ribonucleic acid (HCV RNA), or human immunodeficiency virus (HIV) • History of another primary malignancy except for malignancies treated with curative intent and no known active disease ≥3 years before first dose of study medication • Any co-existing medical condition that in the investigator’s judgement will substantially increase the risk associated with the patient’s participation in the study.

### Study procedures

Before a patient’s involvement in the clinical study, the investigator is under obligation to obtain written informed consent. The randomization will be done by a validated program using variance minimization method [17]. All randomization design aspects and patient allocation statuses underly strict access control. The allocation of the patient numbers and treatment groups will be coordinated by an Interactive Web Response System (IWRS) system integrated in the eCRF. After 1:1 randomization, eligible patients will receive either atezolizumab (1200 mg fixed dose, every 3 weeks [Q3W]) and TRT (30 Gy in 10 fractions) in arm A or atezolizumab only (1200 mg fixed dose, Q3W) in arm B. Patients will be stratified during randomization according to presence of brain metastases (yes vs no), thoracic response after induction therapy (complete/partial response [CR/ PR] vs stable disease [SD]), and prophylactic cranial irradiation PCI (yes vs no). Treatment will be discontinued in case of progressive disease, unacceptable toxicity, on patients’ request, or at the end of study.

In order to ensure patient safety with regard to a potential increase in risk of treatment-related pneumonitis when combining TRT with immune checkpoint inhibition, a safety interim analysis will be performed in arm A after *n =* 23 patients have been followed for 3 months after the end of TRT. If in this cohort the number of patients with a grade ≥ 3 pneumonitis is 2 or more, recruitment to the trial will be stopped. All evaluations will be performed by the medically responsible experts of this trial, i.e. the Coordinating Investigator, the Deputy Coordinating Investigator and the Mentoring Coordinating Investigator– under support of the study statistician as far as applicable. They will provide their opinion to the Sponsor or its delegate who will be responsible for the formal decision on termination / continuation. The Safety Monitoring Committee (SMC) will additionally review all evaluations / decisions and provide its opinion to the Sponsor.

During treatment, tumor response will be assessed according to Response Evaluation Criteria in Solid Tumors (RECIST), version 1.1 by radiological imaging by computed tomography (CT) and/or magnetic resonance imaging (MRI) of the chest and upper abdomen at baseline, then every 6 weeks for the first 36 weeks, and every 9 weeks thereafter until occurrence of disease progression, according to the standard of care. After treatment discontinuation for reasons other than progressive disease, imaging will be performed accordingly to the above-mentioned schedule until progression, death, initiation of another anti-cancer therapy according to the standard of care or end of study. After the end of study, subjects will be proactively followed up regarding treatment-related adverse events until resolved, returned to baseline or deemed irreversible, until lost to follow-up, or withdrawal of study consent. All patients will be followed for survival. Follow-up by phone every 3 months (every 12 weeks [Q12W] ± 14 days) will be offered to patients who decline to return to the site for evaluations. The investigators of the study are responsible for the further treatment of the patient after the end of the study treatment for disease progression and shall support and advice the patients.

Safety assessment during this trial will include physical examinations, Eastern Cooperative Oncology Group (ECOG) performance status, clinical laboratory profile and continuous assessments of adverse events. The brief summary of all study procedures is presented in Table 2. An electronic case report form (eCRF) will be filled by the principal investigator or authorized study stuff person for each participant for data collection throughout the entire trial. The investigator must obtain written informed consent before patient participates in the clinical study. In addition, an adjudication committee has been established to exclude interstitial lung disease (ILD) in all pre-screened patients prior to study inclusion. All observed toxicities and side effects will be recorded in the eCRF and graded according to National Cancer Institute Common Terminology Criteria for Adverse Events (NCI-CTCAE) v5.0 for all patients and their relationship to all study treatment/ procedures assessed and summarized.

**Table 2:** Schedule of assessments

Procedure/ Point in Time	Screening	Treatment				Post- Treatment		
	Inclusion	C1D1	Each cycle (q2w ± 3 days)	Every second cycle (q4w ± 3 days)	EOT	Safety FU-1*	Safety FU-2**	FU (q12w ± 14 days)
Central review – ILD exclusion^#^	(x)							
Informed consent, eligibility criteria, demographics, medical and disease history	x							
Prior and Concomitant Medication Review	x	x	x		x			
Allocation/Randomization	x							
Vital Signs, O_2_ Saturation, and Weight	x	x	x		x	x	x	
ECOG Performance Status	x	x	x		x			
Pregnancy Test, CBC with Differential, Serum Chemistry Panel, Thyroid function test	x	x	x		x			
Urinalysis	x	Whenever clinically indicated						
12-lead ECG	x	Whenever clinically indicated						
Pulmonary function tests	x	x^a^		together with staging	x	x	x	
AEs	x	x	x		x	x	x	x
Full Physical Examination	x							
Directed Physical Examination		x	x		x	x	x	
FACT-L questionnaire	x	x		x	x	x	x	(x)
Tumor Imaging	x	(x^b^)		(x^c^)	(x^d^)			(x^d^)
Tissue	x	Optional: Re-Biopsy at time of progression						
Blood and stool	x	(x^e^)	x	x	x			
Atezolizumab administration		x	x					
Radiotherapy (Arm A^f^)		x						

^#^: A sponsor-independent adjudication committee was established to exclude interstitial lung disease (ILD) [Excl. criterion 5] in all pre-screened patients prior to study inclusion. All study sites are encouraged to provide tumor imaging in pseudonymize form during the (pre-)screening phase for the central review process

*: The regular 30-day full safety follow-up period (FU-1) begins on the day after the EOT visit and lasts approximately 30 days. The FU-1 visit occurs at or near the end of the 30-day safety follow-up period (±7 days)

**: The regular 90-day full safety follow-up period (FU-2) begins on the day after the EOT visit and lasts approximately 90 days. The FU-2 visit occurs at or near the end of the 90-day safety follow-up period (±7 days)

^a^: To be performed on C1D1 if in accordance with local standard

^b^: Chest X-ray to be performed on cycle 1 if in accordance with local standard

^c^: First on-study imaging to be performed 6 weeks (± 7 days) after baseline imaging. Further on-study imaging to be performed Q6W (42 days ±7 days) for the first 36 weeks, and every 9 weeks (±7 days) thereafter until occurrence of disease progression, according to the standard of care

^d^: Only applicable if EOT not according to already detected disease progression

^e^: Biomarker sample to be taken prior to first study drug medication or before first administration of thoracic radiotherapy for arm A, either during screening or C1D1 visit

^f^: Patients in arm A receive thoracic radiotherapy with a dose fractionation of 10 × 3 Gy (30 Gy) within 2 weeks (+ 7 days) in combination with atezolizumab treatment. Thoracic radiotherapy to be started within 7 weeks after C4D1 of induction therapy

The Institute for Clinical Cancer Research (IKF) at Northwest hospital in Frankfurt (Germany) will be responsible for data management and data quality assurance following current Standard Operational Procedures (SOPs) of the IKF.

### Radiotherapy

Radiotherapy will be conducted as described in Slotman et al [2]. Radiotherapy planning is based on CT no older than 4 weeks (+ 3 days) prior to radiotherapy initiation. In the radiotherapy arm (Arm A), 10 fractions of 3 Gy single dose will be prescribed (total dose 30 Gy). The volume of both normal lungs, i.e. both lungs minus clinical target volume (CTV) receiving more than 20 Gy, is recommended to be kept < 35%. Gross tumor volumes (GTV), comprising the post-chemo−/immunotherapy volume of both the thoracic primary tumor formation and any lymph node metastasis, diagnosed either histologically or by imaging procedures before initiation of medical treatment (chemotherapy, immunotherapy), such as CT, positron emission tomography (PET) /CT or endoscopically assisted ultrasound, (GTV) will be outlined by the responsible clinician. Clinicians are encouraged to design a CTV by adding a safety margin of 6 – 8 mm to the GTV and additionally include hilar, mediastinal and supraclavicular lymph node regions, if these contain lymph node metastases. To design the planning target volume (PTV), additional margins of 6-10 mm axially and 9 – 15 mm in cranial-caudal direction will be added to account for motion and setup errors. Both lungs, esophagus, heart and spinal cord must be outlined throughout the PTV. Both techniques, 3D-conformal and any intensity-modulated photon radiation technique (IMRT, VMAT/Radio ARC), are acceptable. Radiotherapy must be performed with photons at energies ranging from 6 to 23 MeV. Dose prescription will follow international commission of radiation units and measurements reports (ICRU 50, 62, 83).

According to the German S3-guideline (AWMF-Registernummer: 020/007OL), patients can either undergo PCI or imaging controls with 3-monthly MRI examinations and early initiation of radiation in case of subsequent metastases. PCI, if indicated by the treating clinicians, will be conducted according to institutional SOC. Mask fixation, 3D-CT-planning, and photon irradiation with either 5 × 4 Gy, 10 - 12 × 2.5, or 15 × 2 Gy are recommended. Each center has to preselect one prophylactic irradiation scheme for all patients. It is not a requirement to withhold ate-zolizumab during PCI. PCI and thoracic radiotherapy can be performed in parallel.

### Collection of biomaterials for translational research

An accompanying translational research project will investigate the mechanisms behind potential tumor-specific immune effects that might be induced by the combination of ICI and radiotherapy and will explore potential biomarkers for such a treatment. To this end, blood and stool samples will be obtained at baseline, on the first day of the second and the fourth cycle, and at the time of disease progression. Collection of tumor tissue samples will take place at baseline and is highly recommended in case of a re-biopsy after disease progression under study treatment. While the baseline tissue collection is mandatory, collection of all other biomarker samples is optional, i.e. patients can participate in the clinical trial if they do not consent to the collection of biomarker samples.

### Study endpoints

The primary endpoint of the TREASURE trial is OS defined as time from randomization to death due to any cause. Secondary efficacy endpoints include 1- and 2-year OS rates, PFS, response rate, and the intrathoracic tumor control defined as rate of intrathoracic progression and time to intrathoracic progression. Moreover, safety will be assessed by evaluating the incidence, nature, causal relationship and severity of adverse events according to CTCAE v5.0 (with special focus on pulmonary events including pneumonitis; hepatic, gastrointestinal, endocrine, ocular, dermatologic, renal and pancreatic events including pancreatitis; immune-mediated myocarditis and myositis as well as infusion-related reactions and cytokine-release syndrome) and the frequency of abnormal laboratory parameters, and feasibility will be addressed in terms of frequency of treatment withdrawal, i.e. due to adverse events or other reasons, and completion of radiotherapy. Furthermore, cancer-related quality of life will be measured using the Functional Assessment of Cancer Therapy – Lung (FACT-L) questionnaire. The planned biomarker analysis of biomaterials that will be collected during the clinical trial is an exploratory endpoint of the TREASURE study.

### Statistical analysis

Statistical analysis is in accordance with the International Conference on Harmonization (ICH) Guidelines “Structure and Content of Clinical Study Reports” and “Statistical Principles for Clinical Trials”. All statistical analyses will be done according to the current SOPs of the Institute of Medical Biometry and Informatics (IMBI), University of Heidelberg, using SAS version 9.4 or higher.

#### Sample size calculation

With *n =* 104 patients, under the assumption of a 20% improvement (from 52 to 72%) in the 12-month OS rate, a difference between treatment groups with regard to the primary endpoint OS can be detected with a power of 80% using a log-rank test at a two-sided alpha of 5%, assuming a dropout rate of about 10%, follow-up phase of 24 months, and an accrual period of 24 months. This improvement of 20% points corresponds to the effect of the radiation therapy together with the one mediated by ICI [2, 6]. Based on these assumptions, the resulting total sample size yielding the necessary number of 66 events is 92 patients (46 per arm) to achieve a power of 80%. In order to adjust for dropouts, *n =* 104 patients have to be randomized.

#### Interim safety analysis

A safety interim analysis will be performed in Arm A after *n =* 23 patients in this arm have been followed for 12 weeks after the end of thoracic radiotherapy. With a sample size of *n =* 23, it will be possible to distinguish between two scenarios for the safety variable:

1. a toxicity scenario, where the grade ≥ 3 pneumonitis rate is assumed to be pTox = 0.125 (based on NICOLAS trial [18] and own unpublished data).

2. a non-toxicity scenario, where the grade ≥ 3 pneumonitis rate is assumed to be pTox = 0.02 (based on IMPower133 [5] and data from trials with other programmed death-1 [PD-1]/PD-L1 inhibitors [19–22]).

If among 23 patients the number of patients with a grade ≥ 3 pneumonitis (X) is 2 or more, no further patients will be recruited to the trial.

Under the above assumptions, the probability to correctly detect the toxicity scenario amounts to P [X ≥ 2|pTOX = 0.125] = 0.801, while the probability to correctly continue amounts to P [X ≤ 1|pTOX = 0.02] = 0.923, assuming binomially distributed variables. Assuming that both scenarios are equally likely, this will yield an overall correct decision probability of 0.862.

#### Methods of statistical analysis

The primary efficacy analysis will be based on the intention-to-treat (ITT) population. Sensitivity analyses of the primary endpoint will be based on the per-protocol (PP) set. The primary endpoint will be analyzed by performing multivariable cox-regression adjusting for the variable therapy group and the stratification variables. The two-sided significance level is set to α = 0.05 (two-sided). Secondary endpoint analyses will be performed descriptively. Secondary endpoint analyses for group comparison include t-tests and chi-squared tests for continuous and ordinal or dichotomous variables, respectively. Feasibility will be analyzed by a description of absolute and relative frequencies of treatment withdrawal, which will be compared between treatment groups by chi-squared tests. Safety analysis will be done for all of the patients who received at least one dose of study medication and will comprise a description of relative and absolute frequencies of adverse events, severity grade based on the CTCAE Version 5.0. The adverse event summary tables will provide the number and percentage of patients with adverse events and the 95% Clopper-Pearson type confidence intervals for the event rates.

### Trial status

As of January 2022, 19 study sites (18 in Germany, 1 in Austria) are initiated. The first patient was enrolled on 28th July 2020. The TREASURE trial is currently recruiting patients.

## Discussion

Despite recent advances in therapeutic landscape of treatment of SCLC, survival rates are still poor and new treatment options are required. The phase II TREASURE study is designed to examine efficacy and safety of the addition of TRT to atezolizumab maintenance therapy in patients who have responded to an induction chemo-immunotherapy.

The Phase II DETERRED trial has demonstrated safety of combining atezolizumab with thoracic radiation in NSCLC patients at even higher total doses than in the current TREASURE protocol [13], and other trials in different solid tumors also support that combination of immunotherapeutic drugs and radiotherapy is safe and well tolerated [14–16]. However, there is still a certain overlap in toxicities observed in the combination treatment. As numerous clinical investigations have shown that dosimetric parameters, such as V20, V30, and mean lung dose, can reduce the risk of lung damage, the proposed radiation protocol applied in this trial has been designed accordingly to reduce the risk of pneumonitis events induced by thoracic radiation therapy. Additionally, in order to enroll only patients with sufficient pulmonary resources to persevere a pneumonitis, inclusion and exclusion criteria include cut-offs for pulmonary function and exclude oxygen-dependent patients from this trial. Nevertheless, considering the possible risk of a higher rate of pneumonitis events in the combinatorial treatment group, a safety interim analysis will be performed in this arm. Furthermore, an accompanying translational research program will address questions regarding the effect of radiotherapy on the immunological status of the tumor and will explore biomarkers, which are much needed for checkpoint blockade with and without concurrent radiotherapy in SCLC patients. In summary, the TREASURE trial will help to gain deeper insight into the interplay between immunotherapy and radiotherapy, and, thus, extend the therapeutic options for ED SCLC patients. Patient recruitment for the TREASURE trial started in September 2020, and participating facilities across Germany and Austria are currently enrolling patients who match the eligibility criteria.

## Data Availability

Data generated by this study will be available for access from the corresponding author upon reasonable request.

## Abbreviations

AE: Adverse event
AIO: Arbeitsgemeinschaft Internistische Onkologie
CR: Complete response
CRF/ eCRF: Case report form / electronic case report form
CRT: Chemoradiation therapy
CT: Computed tomography
CTCAE: Common terminology criteria for adverse events
CTV: Clinical target volume
ECG: Electrocardiogram
ECOG: Eastern Cooperative Oncology Group
ED: Extensive disease
EOT: End of treatment
FACT-L: Functional Assessment of Cancer Therapy - Lung
FU: Follow-up
GTV: Gross tumor volume
Gy: Gray
HR: Hazard ratio
ICI: Immune checkpoint inhibitors
ILD: Interstitial lung disease
ISMB: Independent safety monitoring board
ITT: Intention-to-treat
IWRS: Interactive Web Response System
MHC I: Major histocompatibility complex
MRI: Magnetic resonance imaging
NSCLC: Non-small cell lung cancer
OS: Overall survival
PCI: Prophylactic cranial irradiation
PD-1: Programmed death-1
PD-L1: Programmed death- ligand 1
PET: Positron emission tomography
PFS: Progression-free survival
PP: Per-protocol
PR: Partial response
PTV: Planning target volume
Q3W: Every 3 weeks
Q12W: Every 12 weeks
R: Randomization
RECIST 1.1: Response evaluation criteria in solid tumors, version 1.1
SAE: Severe adverse event
SCLC: Small cell lung cancer
SD: Stable disease
SMC: Safety Monitoring Committee
TRT: Thoracic radiotherapy
